# Neutropenic sepsis is associated with distinct clinical and biological characteristics: a cohort study of severe sepsis

**DOI:** 10.1186/s13054-016-1398-y

**Published:** 2016-07-18

**Authors:** John P. Reilly, Brian J. Anderson, Kristin M. Hudock, Thomas G. Dunn, Altaf Kazi, Anna Tommasini, Dudley Charles, Michael G. S. Shashaty, Mark E. Mikkelsen, Jason D. Christie, Nuala J. Meyer

**Affiliations:** Division of Pulmonary, Allergy, and Critical Care, University of Pennsylvania, Perelman School of Medicine, 3400 Spruce Street, Philadelphia, 19104 PA USA; Center for Clinical Epidemiology and Biostatistics, University of Pennsylvania, Perelman School of Medicine, Philadelphia, PA USA; Division of Pulmonary, Critical Care, and Sleep Medicine, University of Cincinnati, Cincinnati, OH USA; Division of Pulmonary Biology, Cincinnati Children’s Hospital Medical Center, Cincinnati, OH USA

**Keywords:** Neutropenia, Sepsis, Critical illness, Septic shock, Immunocompromised host, Inflammation

## Abstract

**Background:**

Immunocompromised patients who develop sepsis while neutropenic are at high risk for morbidity and mortality; however, it is unknown if neutropenic sepsis is associated with distinct clinical and biological characteristics.

**Methods:**

We conducted a prospective cohort study of patients admitted to the medical intensive care unit of an academic medical center with severe sepsis. Patients were followed for the development of acute respiratory distress syndrome (ARDS), acute kidney injury (AKI), and mortality. Plasma proteins, representing the host inflammatory response, anti-inflammatory response, and endothelial leak were measured in 30 % of subjects. Clinical characteristics and plasma protein concentrations of patients with neutropenia at enrollment were compared to patients without neutropenia.

**Results:**

Of 797 subjects enrolled, 103 (13 %) were neutropenic at ICU admission. The neutropenic subjects were more often in shock, admitted from the hospital ward, had higher APACHE III scores, and more likely bacteremic. Neutropenia was an independent risk factor for AKI (RR 1.28; 95 % CI 1.04, 1.57; *p* = 0.03), but not ARDS (RR 0.90; 95 % CI 0.70, 1.17; *p* = 0.42) or 30-day mortality (RR 1.05; 95 % CI 0.85, 1.31; *p* = 0.65). Neutropenic subjects had higher plasma interleukin (IL)-6 (457 vs. 249 pg/ml; *p* = 0.03), IL-8 (581 vs. 94 pg/ml; *p* <0.001), and granulocyte colony-stimulating factor (G-CSF) (3624 vs. 99 pg/ml; *p* <0.001). Angiopoietin-2 and IL-1 receptor antagonist concentrations did not differ between groups.

**Conclusions:**

Neutropenic sepsis is associated with a higher AKI risk and concentrations of inflammatory mediators IL-6, IL-8, and G-CSF relative to non-neutropenic patients. These differences may have implications for future therapies targeting neutropenic sepsis.

**Electronic supplementary material:**

The online version of this article (doi:10.1186/s13054-016-1398-y) contains supplementary material, which is available to authorized users.

## Background

The incidence of severe sepsis is increasing among hospitalized patients in the developed world [1, 2], while therapeutic options beyond supportive care remain limited [3, 4]. Currently, sepsis is the leading cause of death in non-cardiac intensive care units (ICUs) [5]. The host response to systemic infection is heterogeneous, influenced by clinical and molecular factors, resulting in variability in the pathogenic processes of sepsis and its outcomes. Immunocompromise caused by cancer, immunodeficiency, or immunosuppressing therapy is increasingly common in ICUs and is a strong contributor to sepsis risk [6]. Furthermore, immunocompromised states are associated with lower sepsis survival [7], with neutropenia being a particularly high-risk condition for critically ill patients with sepsis [8]. As the neutrophil is believed to have a central role in the pathogenesis of sepsis and related organ dysfunction [9, 10], understanding whether neutropenic patients with sepsis demonstrate distinct clinical or molecular characteristics is an important yet unanswered question.

In this single-center prospective cohort study of critically ill subjects with severe sepsis, we sought to determine if neutropenia is associated with distinct clinical and biological factors compared to patients without neutropenia, as heterogeneity in host response to infection is likely a strong determinant of sepsis risk, mortality, and potentially response to experimental therapy. To accomplish this objective, we examined clinical characteristics, outcomes, and plasma biomarker profiles among neutropenic and non-neutropenic subjects with severe sepsis. We selected plasma biomarkers, measured at ICU admission, known to be associated with sepsis outcomes, including interleukin (IL)-6, IL-8, granulocyte colony-stimulating factor (G-CSF), angiopoietin-2 (ANG2), and IL-1 receptor antagonist (IL1RA). IL-6 and IL-8 are inflammatory markers most strongly associated with severity of sepsis and death [11–13]. Plasma G-CSF partially regulates IL-8 and IL-6 production and is an important stimulating factor for granulocyte production [14, 15]. ANG2 is a marker of activated endothelium, results in increased vascular permeability [16], and is associated with sepsis severity, shock, organ dysfunction, and death [17–19]. Plasma interleukin-1 receptor antagonist (IL1RA) is an inhibitory anti-inflammatory cytokine that antagonizes the proinflammatory effects of IL-1 alpha and beta at the IL-1 receptor and has been associated with septic shock, acute respiratory distress syndrome (ARDS), and multi-organ failure [20, 21]. By measuring these select plasma proteins, we aimed to characterize the relative contributions of inflammatory/anti-inflammatory signaling and endothelial permeability to neutropenic sepsis. If neutropenic sepsis is associated with distinct clinical or biological variables, this knowledge should inform future tests of emerging precision medicine options in this population [22].

## Methods

### Study population

Patients presenting to the medical ICU at the Hospital of the University of Pennsylvania, a large urban tertiary-care referral center with active cancer, solid organ transplant, and bone marrow transplant programs, were screened for sepsis. Patients who met the American College of Chest Physicians/Society of Critical Care Medicine consensus criteria for severe sepsis or septic shock [23] were eligible for participation in the Molecular Epidemiology of Severe Sepsis in the ICU (MESSI) cohort study if infection-related organ dysfunction was deemed the primary cause for ICU admission [24]. Patients were excluded for lack of commitment to life support at the time of ICU admission, previous enrollment in MESSI, or admission from a long-term care facility. All patients enrolled from cohort initiation in September 2008 until February 2013 were included in the current study. The Institutional Review Board of the University of Pennsylvania approved the study with a waiver of timely informed consent. Informed consent was subsequently obtained from subjects or their proxies, who could withdraw at any time.

### Data collection

Clinical and laboratory data of prospectively enrolled subjects were collected from the electronic medical record by trained research personnel, using structured case report forms. Data collected included baseline demographics, chronic health information, and physiologic, microbiologic, and laboratory data. Race was self-reported by the patient or proxy.

### Exposure and outcome definitions

Neutropenia was defined as an absolute neutrophil count (ANC = polymorphonuclear leukocytes + band forms) less than 1000/microliter on manual or automated differential on the day of ICU admission in the presence of an immunocompromising condition [25]. Immunocompromising conditions included solid malignancies with recent cytotoxic medication administration, hematologic malignancies (i.e., leukemia, lymphoma, or myeloma) including individuals who had undergone hematopoietic stem cell transplantation, the acquired immune deficiency syndrome (AIDS), solid organ transplantation, conditions resulting in bone marrow failure including aplastic anemia, primary or congenital immunodeficiency, or use of cytotoxic medications for a chronic condition, such as a rheumatologic disease. In order to ensure that neutropenia predated sepsis and was not the result of sepsis, we excluded patients without a known or subsequently diagnosed immunocompromising condition who were neutropenic on ICU presentation.

Patients were followed for 6 days after ICU admission for the development of organ dysfunction, specifically, ARDS and acute kidney injury (AKI). ARDS was defined in accordance with the Berlin definition with the added requirement of invasive mechanical ventilation [26]. Arterial blood gases were obtained from the medical record and chest radiographs were interpreted by two physician investigators (JPR, NJM), trained in standardized interpretation for ARDS. Acute kidney injury (AKI) was determined by Acute Kidney Injury Network (AKIN) creatinine and renal replacement therapy consensus criteria [27], using all creatinines ordered for clinical purposes over the first 6 days of ICU admission. Mortality was defined at 30 and 60 days.

### Plasma collection and analysis

Enzyme-linked immunosorbent assays (ELISA) were performed on citrated plasma drawn on the day of ICU admission. ELISA analyses were performed on 245 subjects (30 %) due to limitations of plasma volume, availability, and cost. The following plasma proteins were measured individually by commercially available ELISA kits optimized for human plasma: IL-8, IL-6, ANG2, G-CSF, and IL1RA (R&D Systems, Minneapolis, MN, USA). Please see online supplement (Additional file 1: Table S1) for limits of detection, observed range, and intra-individual coefficients of variation for each analyte.

### Statistical analysis

Clinical characteristics were compared between neutropenic and non-neutropenic patients using the Pearson’s chi-square or Fisher’s exact test for categorical data and the Student’s *t* or Wilcoxon rank-sum test for continuous data. Plasma protein concentrations were compared using the Wilcoxon rank-sum test, and additional analyses were conducted stratified by ICU admission source. We also tested for an association between neutropenia at ICU admission and ARDS, AKI, and mortality using multivariable logistic regression. Possible confounders associated with the exposure or outcome with *p* <0.20 were considered for inclusion in adjusted models, including severity of illness as measured by the Acute Physiology and Chronic Health Evaluation III (APACHE III) score. In adjusted analyses, the white blood cell count and immunocompromising condition components of the APACHE III score were removed as these variables help define the exposure (neutropenia). Additionally, blood gas components and renal components were removed from APACHE III in adjusted analyses for the outcomes ARDS and AKI, respectively. Based on a final sample size of 794 subjects and an expected incidence of ARDS, AKI, and mortality of 40 %, we estimated we would have 80 % power to detect a 15 % absolute difference in outcomes. Spearman correlation coefficients were calculated to test for co-linearity of plasma analyte pairs. Additionally, sensitivity analyses were performed including only non-neutropenic patients with an immunocompromising condition as the comparison group. Sensitivity analyses were also conducted considering immunocompromising condition as the exposure variable rather than neutropenia to assess whether clinical or biomarker differences were attributable to neutropenia or immunocompromise. All analyses were performed in Stata v.12 (StataCorp, College Station, TX, USA).

## Results

### Clinical characteristics

**Fig. 1:**
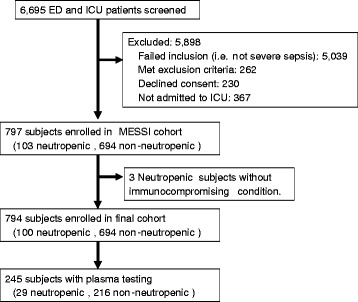
Screening and enrollment. *ED* emergency department, *ICU* intensive care unit, *MESSI* Molecular Epidemiology of Severe Sepsis in the ICU

The MESSI cohort enrolled 797 critically ill subjects with severe sepsis or septic shock over the enrollment period, 103 (13 %) of who were categorized as neutropenic at ICU admission (Fig. 1). The septic subjects had a mean age of 60 +/− 16 and APACHE III score of 77 +/− 24. Forty-three percent were female, with a race distribution of 57 % white, 34 % black, and 2 % Asian. Septic shock was present in 62 % of subjects, and pneumonia was the most common source of sepsis (42 %). Three neutropenic subjects did not possess a predisposing immunocompromising condition, had transient neutropenia (<24 hours), and were removed from all further analyses. Among the neutropenic subjects, the immunocompromising condition was most commonly a hematologic malignancy (n = 74, 74 %), followed by a solid malignancy (n = 14, 14 %), aplastic anemia (n = 4, 4 %), solid organ transplantation (n = 3, 3 %), a rheumatologic condition (n = 1, 1 %, granulomatosis with polyangiitis), and AIDS (n = 1, 1 %). Three subjects (3 %) had more than one immunocompromising condition, including two subjects with solid malignancies and treatment-related acute myeloid leukemia, and one subject with AIDS and lung cancer. The median ANC at enrollment for neutropenic subjects was 44 (IQR, 0–295) cells per microliter and for non-neutropenic subjects was 10,510 (IQR, 6225–16,025) cells per microliter.

Compared to patients without neutropenia, the neutropenic subjects were slightly younger with a higher severity of illness, more often Caucasian, more likely to have bacteremia, more often admitted from the hospital ward rather than the emergency department (ED), more likely to have a blood or vascular source of infection (e.g., catheter-related bloodstream infection), and more likely to be in shock on admission to the ICU (Table 1). Neutropenic patients were less likely to have diabetes, chronic kidney disease, congestive heart failure, or chronic liver disease. Neutropenic subjects were significantly more likely to develop AKI (58 % vs. 28 %, *p* <0.01) and had a significantly higher mortality at 30 days (53 % vs. 41 %, *p* = 0.02) (Table 1); however, after adjustment for confounders including severity of illness and admission source, neutropenia was independently associated with AKI but not mortality (Table 2). The risk of incident ARDS within 6 days of ICU admission for sepsis was not significantly different between groups in adjusted or unadjusted analyses (Tables 1 and 2). Analyses limiting the control group to patients with an immunocompromising condition (n = 252 in the non-neutropenic group) did not significantly alter any results (Additional file 1: Table S2 and Table S3). Possessing an immunocompromising condition alone was not associated with ARDS or AKI, but was associated with increased mortality (Additional file 1: Table S4).

**Table 1:** Patient characteristics by neutropenia status^*a*^

Patient characteristic	Non-neutropenic (n = 694)	Neutropenic (n = 100)	*p* ^*b*^
Age	60 ± 16	58 ± 12	0.08
Male sex	387 (56 %)	66 (66 %)	0.06
Race			
White	380 (55 %)	73 (73 %)	<0.01
Black	255 (37 %)	17 (17 %)	
Asian	19 (3 %)	0 (0 %)	
Other/unknown	40 (6 %)	10 (10 %)	
APACHE III	74 (59–90)	87 (71–109)	<0.01
Documented bacteremia	182 (26 %)	43 (43 %)	<0.01
Source of infection			
Pulmonary	289 (42 %)	40 (40 %)	0.05
Genitourinary	82 (12 %)	6 (6 %)	
Abdominal/gastrointestinal	93 (13 %)	15 (15 %)	
Head/neck	17 (2 %)	0 (0 %)	
Blood^*c*^	58 (8 %)	15 (15 %)	
Skin/soft tissue/bone	40 (6 %)	2 (2 %)	
Gynecologic	2 (0 %)	0 (0 %)	
Unclear source	113 (16 %)	22 (22 %)	
Shock at presentation	371 (53 %)	74 (74 %)	<0.01
Aminoglycoside exposure	182 (26 %)	65 (67 %)	<0.01
IV contrast exposure	143 (21 %)	23 (23 %)	0.97
ICU admitting source			
Emergency department	436 (63 %)	33 (33 %)	<0.01
Hospital ward	200 (29 %)	64 (64 %)	
Other institution	58 (8 %)	3 (3 %)	
Comorbidities			
Lymphoma	45 (6 %)	13 (13 %)	0.02
Leukemia	33 (5 %)	52 (52 %)	<0.01
Multiple myeloma	21 (3 %)	10 (10 %)	<0.01
Solid malignancy	82 (12 %)	16 (16 %)	0.24
Solid organ transplant	50 (7 %)	3 (3 %)	0.08
AIDS	11 (2 %)	2 (2 %)	0.67
Diabetes	225 (32 %)	19 (19 %)	<0.01
Chronic renal disease	119 (17 %)	9 (9 %)	0.04
Congestive heart failure	107 (15 %)	6 (6 %)	0.01
Chronic liver disease	81 (12 %)	2 (2 %)	<0.01
Outcomes			
ARDS^*d*^	267 (38 %)	42 (42 %)	0.51
AKI^*d*^	263 (38 %)	58 (58 %)	<0.01
30-day mortality	285 (41 %)	53 (53 %)	0.02
60-day mortality	311 (45 %)	60 (60 %)	<0.01

*APACHE* Acute Physiology and Chronic Health Evaluation, *IV* intravenous, *ICU* intensive care unit, *AIDS* acquired immune deficiency syndrome, *ARDS* acute respiratory distress syndrome, *AKI* acute kidney injury

^*a*^Data are shown as n (%) for categorical variables, mean ± standard deviation for normally distributed continuous variables, and median (interquartile range) for non-normally distributed continuous variables

^*b*^Normally distributed continuous variables were compared using the Student’s *t* test, non-normally distributed continuous variables using the Wilcoxon rank-sum test, and categorical variables using a Pearson chi-square test or Fisher’s exact test

^*c*^Blood source of infection included catheter-related blood stream infections, endocarditis, and thrombophlebitis

^*d*^Patients were followed for the outcomes ARDS and AKI over the first 6 days of ICU admission

**Table 2:** Associations of neutropenia with acute respiratory distress syndrome risk, acute kidney injury risk, and mortality

	Standardized risk, % (95 % CI)^*a*^					
Outcomes	Neutropenic	Non-neutropenic	Unadjusted RR^*b*^ (95 % CI)	*p*	Adjusted RR^*b*^ (95 % CI)	*p*
ARDS^*c*^	36 % (27, 44 %)	40 % (36, 43 %)	1.09 (0.85, 1.40)	0.51	0.90 (0.70, 1.17)	0.42
AKI^*d*^	54 % (44, 64 %)	42 % (38, 46 %)	1.39 (1.15, 1.69)	0.002	1.28 (1.04, 1.57)	0.03
30-day mortality^*e*^	44 % (36, 53 %)	42 % (39, 46 %)	1.29 (1.05, 1.58)	0.03	1.05 (0.85, 1.31)	0.65
60-day mortality^*e*^	52 % (43, 61 %)	46 % (43, 49 %)	1.34 (1.12, 1.60)	0.004	1.13 (0.93, 1.37)	0.23

*CI* confidence interval, *RR* relative risk, *ARDS* acute respiratory distress syndrome, *AKI* acute kidney injury, *APACHE* acute physiology and chronic health evaluation

^*a*^Standardized risks and 95 % confidence intervals by neutropenic status were determined using post-estimation marginal analyses of adjusted multivariable logistic regression models

^*b*^RR and 95 % confidence intervals were estimated using post-estimation marginal analyses of logistic regression models

^*c*^Final ARDS logistic regression models were adjusted for age, sex, race, source of sepsis, admission source, history of chronic renal insufficiency, history of chronic liver disease, and APACHE III without immunocompromising conditions, white blood cell, and arterial blood gas components

^*d*^Final AKI logistic regression models were adjusted for age, sex, race, source of sepsis, admission source, history of diabetes mellitus, history of congestive heart failure, history of chronic renal insufficiency, history of chronic liver disease, and APACHE III without immunocompromising conditions, white blood cell, and renal components

^*e*^Final mortality logistic regression models were adjusted for age, sex, race, source of sepsis, admission source, history of diabetes mellitus, history of congestive heart failure, history of chronic renal insufficiency, history of chronic liver disease, and APACHE III without immunocompromising conditions and white blood cell components

### Plasma protein analysis

**Fig. 2:**
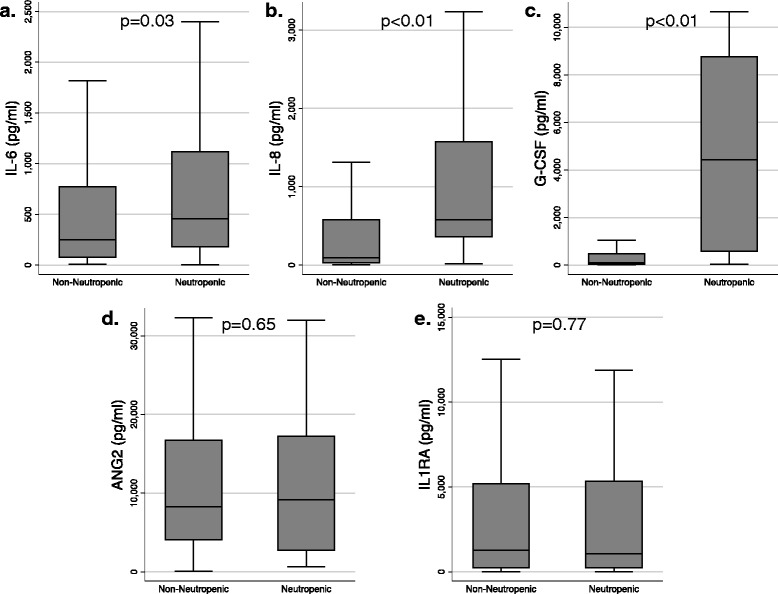
Box and whisker plots comparing plasma (**a**) IL-6, (**b**) IL-8, (**c**) G-CSF, (**d**) ANG2, and (**e**) IL1RA concentration between neutropenic patients and non-neutropenic patients. *IL* interleukin, *G*-*CSF* granulocyte colony-stimulating factor, *ANG2* angiopoietin 2, *IL1RA* interleukin-1 receptor antagonist

Characteristics of the 245 subjects with plasma protein testing (Additional file 1: Table S5) were largely similar to the overall cohort but with slightly lower severity of illness and mortality than the overall population, particularly among the neutropenic plasma subpopulation. Plasma concentrations of IL-6, IL-8, IL1RA, ANG2, and G-CSF are shown in Table 3 stratified by neutropenic status. Subjects with neutropenia had significantly higher plasma IL-6 and IL-8 levels than non-neutropenic subjects (Fig. 2a-b). Plasma IL1RA and ANG2 levels did not vary by neutropenic status (Fig. 2d-e). Because both IL-8 and, to a lesser extent, IL-6 are regulated by G-CSF, we measured G-CSF to contextualize the very high IL-8 levels. Plasma G-CSF concentrations were significantly higher in neutropenic subjects (Fig. 2c). Additionally, G-CSF demonstrated strong correlations with IL-8 (ρ = 0.55, *p* <0.001) and IL-6 (ρ = 0.46, *p* <0.001). Of the 245 subjects with plasma protein measurements, 20 received exogenous G-CSF (filgrastim or pegfilgrastim) within the prior 30 days (16 neutropenic patients and 4 non-neutropenic). Exogenous G-CSF was associated with higher levels of IL-8 (*p* <0.001) and G-CSF (*p* <0.001), but not the other measured analytes. Excluding these 20 subjects from analyses did not change any of the identified associations between neutropenia and plasma protein concentration (Table 3). Analyses limiting the control group only to non-neutropenic subjects with an immunocompromising condition demonstrated similar results (Additional file 1: Table S6). Biomarker concentrations did not differ between immunocompromised patients and non-immunocompromised (Additional file 1: Table S7). Stratified analyses by ICU admission source (i.e., ED, hospital ward, or outside hospital transfer) also demonstrated similar associations between plasma proteins and neutropenia.

**Table 3:** Plasma protein concentrations by neutropenia status^*a*^

	Total population			No exogenous G-CSF^*b*^		
Plasma protein	Non-neutropenic (n = 216)	Neutropenic (n = 29)	*p* ^*c*^	Non-neutropenic (n = 212)	Neutropenic (n = 13)	*p* ^*c*^
ANG2 (pg/ml)	8242 (4039, 16694)	9165 (2708, 17244)	0.65	8252 (3918, 16694)	9475 (3168, 18936	0.68
IL1RA (pg/ml)	1269 (243, 5202)	1049 (103, 5329)	0.77	1346 (249, 3205)	534 (198, 3717)	0.52
IL-6 (pg/ml)	249 (76, 752)	457 (181, 1117)	0.03	249 (75, 773)	457 (281, 1117)	0.11
IL-8 (pg/ml)	94 (27, 600)	581 (358, 1576)	<0.01	93 (27, 600)	478 (361, 1681)	<0.01
G-CSF (pg/ml)	99 (29, 474)	3624 (919, 10099)	<0.01	90 (33, 331)	6660 (3411, 9045)	<0.01

*G*-*CSF* granulocyte colony-stimulating factor, *ANG2* angiopoietin 2, *IL1RA* interleukin-1 receptor antagonist, *IL*-*6* interleukin-6, *IL*-*8* interleukin-8

^*a*^Data are displayed as median (interquartile range)

^*b*^Excluding subjects who received exogenous G-CSF within 30 days of enrollment

^*c*^Plasma protein levels were compared between neutropenic and non-neutropenic using the Wilcoxon rank-sum test

## Discussion

Our study provides evidence that neutropenic sepsis is associated with distinct clinical and molecular factors compared to non-neutropenic sepsis. These findings suggest differences in underlying sepsis pathogenesis, which may require targeted therapies particular to this patient population. Specifically, neutropenic patients with severe sepsis were more likely to present to the ICU in shock and were more likely to have documented bacteremia. Neutropenia at presentation to the ICU was independently associated with a higher risk of AKI, but not 30-day mortality or ARDS within 6 days of ICU admission after adjustment for clinical confounders. Neutropenic patients also displayed higher levels of the inflammatory cytokines IL-6 and IL-8 despite having lower circulating immune cells. Our results support the hypothesis that the pathogenesis of sepsis and related organ dysfunction is different in neutropenic patients, as well as the conclusion that traditional biomarkers of sepsis risk and prognosis may not perform equally in neutropenic and non-neutropenic populations.

Our findings of higher IL-8, IL-6, and G-CSF among neutropenic subjects are consistent with the current knowledge of regulated neutrophil homeostasis, and suggest that the elevations represent a physiologic state whereby tissues are signaling for neutrophil recruitment to fight infection. IL-8 is a neutrophil chemotactic factor [28, 29], while IL-6 stimulates neutrophil production [30, 31]. Typically antigen-presenting cells phagocytose senescent neutrophils and downregulate further granulopoiesis [29]. The lack of senescent neutrophils in neutropenic patients circumvents this feedback inhibition regulating neutrophil balance. This may result in consistently high levels of G-CSF, and subsequently IL-8 and IL-6, in neutropenic subjects. Consistent with this paradigm, we observed strong correlation of G-CSF with IL-8 and IL-6 in our subjects. It is unclear whether elevated G-CSF, IL-8, and IL-6 contribute to the organ dysfunction or increased shock observed in neutropenic sepsis, but these elevations in inflammatory mediators may be maladaptive. Both IL-8 and IL-6 are strongly associated with mortality, AKI, and ARDS in observational studies of sepsis [11–13, 32–34]. IL-6-deficient mice are resistant to AKI in ischemia-reperfusion and nephrotoxin models [35, 36], suggesting IL-6 may have toxic effects on the kidney. Interleukin-8 and its receptors have likewise been suggested to mediate kidney injury in an inflammatory model [37]. Further mechanistic investigation is warranted to test whether high IL-8 and IL-6 levels can mediate kidney injury in the setting of neutropenia, and explain our findings of increased AKI risk among neutropenic patients with sepsis. Alternatively, neutropenic patients may be exposed to more nephrotoxic drugs, including aminoglycosides and/or chemotherapeutics, proceeding or concurrent with sepsis, and the combination of these insults may have predisposed to AKI.

The risk of ARDS within 6 days of ICU admission was similar among the neutropenic and non-neutropenic patients, despite a higher proportion with shock among the neutropenic subjects. The neutrophil is believed to be important early in ARDS pathogenesis, and its absence may protect the lung from injury. Kangelaris and colleagues demonstrated increased expression of neutrophil-related genes dominating the whole blood expression pattern of patients with sepsis and ARDS at ICU admission relative to those with sepsis and no ARDS, supporting the importance of neutrophils early in ARDS [38]. However, ARDS has been well described in neutropenic hosts and was present in 42 neutropenic subjects enrolled in our study, suggesting heterogeneity in ARDS pathogenesis based on the presence or absence of circulating neutrophils [39]. Monocyte and alveolar macrophage deactivation in the setting of septic neutropenic ARDS has been described as one mechanism of neutrophil-independent lung injury [40, 41].

While not detected in our study, as the large majority of our subjects were consistently neutropenic during the study period; several previous studies have reported associations between recovery of peripheral neutrophil counts and the onset or deterioration of ARDS [42–45]. Taken in the context of these previous reports, our findings of elevated IL-6, IL-8, and G-CSF in the setting of neutropenic sepsis suggest that the neutropenic host is primed to rapidly recruit neutrophils from the bone marrow to the peripheral tissues upon count recovery. Younger neutrophils are preferentially sequestered in the pulmonary circulation and may be more likely to contribute to endothelial damage than older neutrophils [46]. Therefore, the rapid recruitment of neutrophils to the lung may be maladaptive in the setting of recovery from prolonged neutropenia. Future therapies could potentially target the marked elevations in IL-6 and IL-8, limiting neutrophil recruitment and subsequent lung injury in patients predictably recovering from neutropenia, such as those undergoing cytotoxic chemotherapy.

Our study has several important limitations. The MESSI cohort was derived from a single-center, tertiary-care hospital with an active bone marrow transplant program; therefore, generalizability may be limited to similar populations. We followed patients for the first 6 days of their ICU stay in order to capture ARDS and AKI related to the initial septic episode; however, may have missed ARDS that developed later in the ICU stay, including during neutropenia recovery. Additionally, we may have been underpowered to detect important associations between neutropenia and clinical outcomes. Negative results should be interpreted with caution. The plasma analysis was limited to only 30 % of the cohort, limiting power to detect potential important associations. However, despite this limitation, ours is among the largest multi-marker studies of patients with sepsis, with larger studies typically employing multiplex arrays [47]. Furthermore, we identified several potentially important plasma proteins that differ between neutropenic and non-neutropenic sepsis. We focused our plasma analysis on proteins we hypothesized to be important in differentiating neutropenic and non-neutropenic sepsis, but did not measure mediators of systemic coagulation, cellular adhesion, or markers of epithelial dysfunction. Additional research may demonstrate that these pathways also contribute to neutropenic sepsis. Lastly, our exposure definition relied solely on a peripheral white blood cell count and differential measured at ICU admission, and may not reflect immune cells located peripherally in tissues, or changes in neutrophil count over time, although the majority of our neutropenic subjects were neutropenic for the entire 6 days of ICU observation.

## Conclusions

In conclusion, our data support the hypothesis that severe sepsis results in a distinct inflammatory response in patients with neutropenia relative to those without neutropenia. Neutropenia was independently associated with a higher AKI risk and was characterized by a profile of high IL-6, IL-8, and G-CSF relative to non-neutropenic sepsis. These findings may have implications toward the development of future sepsis and organ dysfunction therapeutics targeting the neutropenic population.

## Abbreviations

AIDS, acquired immunodeficiency syndrome; AKI, acute kidney injury; AKIN, Acute Kidney Injury Network; ANC, absolute neutrophil count; ANG2, angiopoietin-2; APACHE, Acute Physiology and Chronic Health Evaluation; ARDS, acute respiratory distress syndrome; ED, emergency department; ELISA, enzyme-linked immunosorbent assays; G-CSF, granulocyte colony-stimulating factor; ICU, intensive care unit; IL1RA, interluekin-1 receptor antagonist; IL-6, interleukin 6; IL-8, interleukin 8; MESSI, Molecular Epidemiology of Severe Sepsis in the ICU

## Additional file

Additional file 1: Table S1. Characteristics of enzyme-linked immunosorbent assays for plasma proteins. **Table S2.** Patient characteristics by neutropenia status among patients with an immunocompromising condition. **Table S3.** Associations of neutropenia with acute respiratory distress syndrome risk, acute kidney injury risk, and mortality among patients with an immunocompromising condition. **Table S4.** Association of an immunocompromising condition with acute respiratory distress syndrome risk, acute kidney injury risk, and mortality. **Table S5.** Patient characteristics among subjects undergoing biomarker testing. **Table S6.** Plasma protein concentrations by neutropenia status among patients with an immunocompromising condition. **Table S7.** Plasma protein concentrations in patients with an immunocompromising condition versus without an immunocompromising condition. (DOCX 30 kb)
