# Supplementary material for: Neutropenic sepsis is associated with distinct clinical and biological characteristics: a cohort study of severe sepsis
Source: Crit Care. 2016 Jul 18;20:222. doi: 10.1186/s13054-016-1398-y (PMC4950810; doi:10.1186/s13054-016-1398-y)
Supplement: Additional file 1: Table S1. — Characteristics of enzyme-linked immunosorbent assays for plasma proteins. Table S2. Patient characteristics by neutropenia status among patients with an immunocompromising condition. Table S3. Associations of neutropenia with acute respiratory distress syndrome risk, acute kidney injury risk, and mortality among patients with an immunocompromising condition. Table S4. Association of an immunocompromising condition with acute respiratory distress syndrome risk, acute kidney injury risk, and mortality. Table S5. Patient characteristics among subjects undergoing biomarker testing. Table S6. Plasma protein concentrations by neutropenia status among patients with an immunocompromising condition. Table S7. Plasma protein concentrations in patients with an immunocompromising condition versus without an immunocompromising condition. (DOCX 30 kb) [file 13054_2016_1398_MOESM1_ESM.docx]

Additional file 1

Table S1. Characteristics of Enzyme-Linked Immunosorbant Assays for Plasma Proteins

| Plasma Protein | Limit of Detection | Observed Range | Intra-Individual CV |
| --- | --- | --- | --- |
| ANG2 (pg/ml) | 46.9 | 89.5 – 176,000 | 3.95% |
| IL1RA (pg/ml) | 11.3 | 11.3 – 192,594 | 7.61% |
| IL-6 (pg/ml) | 3.125 | 6.37 – 5447.8 | 12.0% |
| IL-8 (pg/ml) | 1.20 | 1.21 – 22,990 | 9.46% |
| G-CSF (pg/ml) | 1.20 | 2.35 – 39,162 | 12.0% |

CV = coefficient of variation, ANG2 = angiopoietin 2, IL1RA = interleukin-1 receptor antagonist, IL-6 = interleukin-6, IL-8 = interleukin-8, G-CSF = granulocyte colony stimulating factor.

| Patient Characteristic | Non-Neutropenic  (n=252) | Neutropenic  (n=100) | *p^b^* |
| --- | --- | --- | --- |
| Age | 60 ± 13 | 58 ± 12 | 0.11 |
| Male Sex | 141 (56%) | 66 (66%) | 0.09 |
| Race  White  Black  Asian  Other/Unknown | 165 (65%)  68 (27%)  8 (3%)  11 (4%) | 73 (73%)  17 (17%)  0 (0%)  10 (10%) | 0.01 |
| APACHE III | 79 (64 – 94) | 87 (71 - 109) | <0.01 |
| Documented Bacteremia | 50 (19%) | 43 (43%) | <0.01 |
| Source of Infection  Pulmonary  Genitourinary  Abdominal/GI  Head/Neck  Blood*^c^*  Skin/Soft tissue/Bone  Gynecologic  Unclear source | 111 (44%)  21 (8%)  31 (12%)  3 (1%)  16 (6%)  13 (5%)  1 (0%)  56 (22%) | 40 (40%)  6 (6%)  15 (15%)  0 (0%)  15 (15%)  2 (2%)  0 (0%)  22 (22%) | 0.20 |
| Shock at Presentation | 135 (54%) | 74 (74%) | <0.01 |
| Aminoglycoside Exposure | 78 (31%) | 65 (67%) | <0.01 |
| IV Contrast Exposure | 63 (25%) | 23 (23%) | 0.43 |
| ICU Admitting Source  Emergency Department  Hospital Ward  Other Institution | 145 (58%)  94 (37%)  13 (5%) | 33 (33%)  64 (64%)  3 (3%) | <0.01 |
| Comorbidities |  |  |  |
| Lymphoma | 45 (18%) | 13 (13%) | 0.27 |
| Leukemia | 33 (13%) | 52 (52%) | <0.01 |
| Multiple Myeloma | 21 (8%) | 10 (10%) | 0.62 |
| Solid Malignancy | 82 (33%) | 16 (16%) | <0.01 |
| Solid Organ Transplant | 50 (20%) | 3 (3%) | <0.01 |
| AIDS | 11 (4%) | 2 (2%) | 0.29 |
| Diabetes | 67 (27%) | 19 (19%) | 0.14 |
| Chronic Renal Disease | 50 (20%) | 9 (9%) | 0.01 |
| Congestive Heart Failure | 107 (15%) | 6 (6%) | 0.01 |
| Chronic Liver Disease | 15 (6%) | 2 (2%) | 0.12 |
| Outcomes |  |  |  |
| ARDS*^d^* | 101 (40%) | 42 (42%) | 0.74 |
| AKI*^d^* | 92 (39%) | 58 (58%) | <0.01 |
| 30-day Mortality | 128 (51%) | 53 (53%) | 0.71 |
| 60-day Mortality | 142 (56%) | 60 (60%) | 0.53 |

Table S2. Patient characteristics by neutropenia status among patients with an immunocompromising condition.*^a^*

APACHE = acute physiology and chronic health evaluation, ICU = intensive care unit, IV = intravenous, AIDS = acquired immune deficiency syndrome, ARDS = acute respiratory distress syndrome, AKI =acute kidney injury.

*^a^*Data are shown as n (%) for categorical variables, mean ± standard deviation for normally distributed continuous variables, and median (interquartile range) for non-normally distributed continuous variables.

*^b^*Normally distributed continuous variables were compared using the Student’s t-test, non-normally distributed continuous variables using the Wilcoxon rank-sum test, and categorical variables using a Pearson chi-square test or Fisher’s exact test.

*^c^*Blood source of infection included catheter related blood stream infections, endocarditis, and thrombophlebitis.

*^d^*Patients were followed for the outcomes ARDS and AKI over the first 6 days of ICU admission.

Table S3. Associations of neutropenia with acute respiratory distress syndrome risk, acute kidney injury risk, and mortality among patients with an immunocompromising condition.*^a^*

|  |  |  |  |  |
| --- | --- | --- | --- | --- |
| Outcomes | Unadjusted RR*^b^* (95% CI) | p | Adjusted RR*^b^* (95% CI) | p |
| ARDS*^c^* | 1.05 (0.80, 1.38) | 0.74 | 0.93 (0.71, 1.23) | 0.61 |
| AKI*^d^* | 1.47 (1.17, 1.85) | 0.002 | 1.44 (1.15, 1.81) | 0.003 |
| 30-day Mortality*^e^* | 1.09 (0.69, 1.74) | 0.71 | 1.08 (0.79, 1.48) | 0.62 |
| 60-day Mortality*^e^* | 1.16 (0.73, 1.86) | 0.53 | 1.14 (0.87, 1.50) | 0.35 |

ARDS = acute respiratory distress syndrome, AKI = acute kidney injury, RR = relative risk, CI = confidence interval, APACHE = acute physiology and chronic health evaluation.

*^a^*Potentially immunocompromising conditions included solid malignancies, hematologic malignancies (i.e. leukemia, lymphoma, or myeloma), the acquired immune deficiency syndrome (AIDS), solid organ transplantation, conditions resulting in bone marrow failure including aplastic anemia, primary or congenital immunodeficiency, or use of cytotoxic medications for a chronic condition, such as a rheumatologic disease.

*^b^*RR and 95% confidence intervals were estimated using post-estimation marginal analyses of logistic regression models.

*^c^*Final ARDS logistic regression models were adjusted for age, sex, race, source of sepsis, admission source, history of chronic liver disease, history of chronic kidney disease, and APACHE III without immunocompromising conditions, white blood cell, and arterial blood gas components.

*^d^*Final AKI logistic regression models were adjusted for age, sex, race, source of sepsis, admission source, history of diabetes mellitus, history of congestive heart failure, history of chronic liver disease, history of chronic kidney disease, and APACHE III without immunocompromising conditions, white blood cell, and renal components.

*^e^*Final mortality logistic regression models were adjusted for age, sex, race, source of sepsis, admission source, history of diabetes mellitus, history of congestive heart failure, history of chronic liver disease, history of chronic kidney disease, and APACHE III without immunocompromising conditions and white blood cell components.

Table S4. Association of an immunocompromising condition with acute respiratory distress syndrome risk, acute kidney injury risk, and mortality.

|  | Standardized Risk, % (95% CI)*^a^* | |  |  |  |  |
| --- | --- | --- | --- | --- | --- | --- |
| Outcomes | Immuno-compromised  (n=352) | Not Immuno-compromised  (n=442) | Unadjusted RR*^b^* (95% CI) | p | Adjusted RR*^b^* (95% CI) | p |
| ARDS*^c^* | 38% (33, 44%) | 40% (36, 45%) | 1.08 (0.91, 1.28) | 0.39 | 0.93 (0.78, 1.11) | 0.42 |
| AKI*^d^* | 42% (36, 47%) | 46% (41, 51%) | 1.05 (0.89, 1.24) | 0.55 | 0.91 (0.77, 1.08) | 0.27 |
| 30-day Mortality*^e^* | 50% (45, 55%) | 36% (32, 41%) | 1.45 (1.23, 1.70) | 0.01 | 1.38 (1.16, 1.65) | 0.01 |
| 60-day Mortality*^e^* | 56% (51, 62%) | 39% (34, 43%) | 1.50 (1.29, 1.74) | 0.01 | 1.46 (1.24, 1.72) | 0.01 |

ARDS = acute respiratory distress syndrome, AKI = acute kidney injury, RR = relative risk, CI = confidence interval, APACHE = acute physiology and chronic health evaluation.

*^a^*Standardized risks and 95% confidence intervals by neutropenic status were determined using post-estimation marginal analyses of adjusted multivariable logistic regression models.

*^b^*RR and 95% confidence intervals were estimated using post-estimation marginal analyses of logistic regression models.

*^c^*Final ARDS logistic regression models were adjusted for age, sex, race, source of sepsis, admission source, history of chronic kidney disease, history of chronic liver disease, and APACHE III without immunocompromising conditions, white blood cell, and arterial blood gas components.

*^d^*Final AKI logistic regression models were adjusted for age, sex, race, source of sepsis, admission source, history of diabetes mellitus, history of congestive heart failure, history of chronic kidney disease, history of chronic liver disease, and APACHE III without immunocompromising conditions, white blood cell, and renal components.

*^e^*Final mortality logistic regression models were adjusted for age, sex, race, source of sepsis, admission source, history of diabetes mellitus, history of congestive heart failure, history of chronic kidney disease, history of chronic liver disease, and APACHE III without immunocompromising conditions and white blood cell components.

Table S5. Patient characteristics among subjects undergoing biomarker testing.*^a^*

| Patient Characteristic | Non-Neutropenic  (n=216) | Neutropenic  (n=29) | *p^b^* |
| --- | --- | --- | --- |
| Age | 60 ± 16 | 55 ± 12 | 0.04 |
| Male Sex | 115 (53%) | 19 (66%) | 0.21 |
| Race  White  Black  Asian  Other/Unknown | 124 (57%)  73 (34%)  8 (4%)  11 (5%) | 22 (76%)  2 (7%)  0 (0%)  5 (17%) | <0.01 |
| APACHE III | 71 (60 – 88) | 80 (70 - 90) | 0.08 |
| Documented Bacteremia | 62 (29%) | 20 (63%) | <0.01 |
| Source of Infection  Pulmonary  Genitourinary  Abdominal/GI  Head/Neck  Blood*^c^*  Skin/Soft tissue/Bone  Gynecologic  Unclear source | 90 (42%)  22 (10%)  27 (13%)  4 (2%)  20 (9%)  11 (5%)  1 (0%)  41 (19%) | 9 (31%)  3 (10%)  4 (14%)  0 (0%)  5 (17%)  1 (3%)  0 (0%)  7 (24%) | 0.81 |
| Shock at Presentation | 127 (60%) | 19 (66%) | 0.54 |
| ICU Admitting Source  ED  Hospital Ward  Other Institution | 132 (61%)  59 (27%)  25 (12%) | 7 (24%)  21 (72%)  1 (4%) | <0.01 |
| Comorbidities |  |  |  |
| Lymphoma | 12 (6%) | 3 (10%) | 0.31 |
| Leukemia | 14 (6%) | 14 (48%) | <0.01 |
| Multiple Myeloma | 4 (2%) | 4 (14%) | <0.01 |
| Solid Malignancy | 28 (13%) | 5 (17%) | 0.53 |
| Solid Organ Transplant | 14 (6%) | 0 (0%) | 0.38 |
| AIDS | 4 (2%) | 0 (0%) | 1.00 |
| Diabetes | 64 (30%) | 6 (21%) | 0.35 |
| Chronic Renal Disease | 40 (19%) | 1 (4%) | 0.06 |
| Congestive Heart Failure | 39 (18%) | 1 (4%) | 0.06 |
| Chronic Liver Disease | 28 (13%) | 2 (7%) | 0.55 |
| Outcomes*^d^* |  |  |  |
| ARDS | 93 (43%) | 11 (38%) | 0.59 |
| AKI | 93 (46%) | 13 (45%) | 0.92 |
| 30-day Mortality | 101 (47%) | 13 (45%) | 0.83 |
| 60-day Mortality | 107 (50%) | 16 (55%) | 0.59 |

APACHE = acute physiology and chronic health evaluation, ICU = intensive care unit, AIDS = acquired immune deficiency syndrome, ARDS = acute respiratory distress syndrome, AKI = acute kidney injury.

*^a^*Data are shown as n (%) for categorical variables, mean ± standard deviation for normally distributed continuous variables, and median (interquartile range) for non-normally distributed continuous variables.

*^b^*Normally distributed continuous variables were compared using the Student’s t-test, non-normally distributed continuous variables using the Wilcoxon rank-sum test, and categorical variables using a Pearson chi-square test or Fisher’s exact test.

*^c^*Blood source of infection included catheter related blood stream infections, endocarditis, and thrombophlebitis.

*^d^*Patients were followed for the outcomes ARDS and AKI over the first 6 days of ICU admission.

Table S6. Plasma protein concentrations by neutropenia status among patients with an immunocompromising condition.*^a^*

|  | Total Population | | |
| --- | --- | --- | --- |
| Plasma Protein | Non-Neutropenic  (n=81) | Neutropenic  (n=29) | *p^b^* |
| ANG2 (pg/ml) | 7270 (4349, 15327) | 9165 (2708, 17244) | 0.97 |
| IL1RA (pg/ml) | 1205 (126, 3683) | 1049 (103, 5329) | 0.73 |
| IL-6 (pg/ml) | 149 (65, 400) | 457 (181, 1117) | <0.01 |
| IL-8 (pg/ml) | 61 (25, 551) | 581 (358, 1576) | <0.01 |
| G-CSF (pg/ml) | 100 (31, 197) | 3624 (919, 10099) | <0.01 |

ANG2 = angiopoietin 2, IL1RA = interleukin-1 receptor antagonist, IL-6 = interleukin-6, IL-8 = interleukin-8, G-CSF = granulocyte colony stimulating factor.

*^a^*Data are displayed as median (interquartile range).

*^b^*Plasma protein levels were compared between neutropenic and non-neutropenic using the Wilcoxon rank-sum test.

Table S7. Plasma protein concentrations in patients with an immunocompromising condition versus without an immunocompromising condition.

| Plasma Protein | Not Immunocompromised  (n=135) | Immunocompromised  (n=110) | *p^b^* |
| --- | --- | --- | --- |
| ANG2 (pg/ml) | 8505 (3806, 17793) | 7641 (3750, 15553) | 0.41 |
| IL1RA (pg/ml) | 1346 (255, 6089) | 1205 (148, 4393) | 0.47 |
| IL-6 (pg/ml) | 368 (92, 970) | 210 (76, 615) | 0.13 |
| IL-8 (pg/ml) | 101 (28, 580) | 188 (34, 877) | 0.19 |
| G-CSF (pg/ml) | 104 (39, 474) | 162 (36, 1994) | 0.10 |

ANG2 = angiopoietin 2, IL1RA = interleukin-1 receptor antagonist, IL-6 = interleukin-6, IL-8 = interleukin-8, G-CSF = granulocyte colony stimulating factor.

*^a^*Data are displayed as median (interquartile range).

*^b^*Plasma protein levels were compared between neutropenic and non-neutropenic using the Wilcoxon rank-sum test.
